# Etiology and Audiological Outcomes at 3 Years for 364 Children in Australia

**DOI:** 10.1371/journal.pone.0059624

**Published:** 2013-03-28

**Authors:** Hans-Henrik M. Dahl, Teresa Y. C. Ching, Wendy Hutchison, Sanna Hou, Mark Seeto, Jessica Sjahalam-King

**Affiliations:** 1 Murdoch Childrens Research Institute, Royal Children’s Hospital, Melbourne, Australia; 2 Department of Pediatrics, University of Melbourne, Melbourne, Australia; 3 National Acoustic Laboratories, Sydney, Australia; 4 HEARing Cooperative Research Centre, Melbourne, Australia; Innsbruck Medical University, Austria

## Abstract

Hearing loss is an etiologically heterogeneous trait with differences in the age of onset, severity and site of lesion. It is caused by a combination of genetic and/or environmental factors. A longitudinal study to examine the efficacy of early intervention for improving child outcomes is ongoing in Australia. To determine the cause of hearing loss in these children we undertook molecular testing of perinatal “Guthrie” blood spots of children whose hearing loss was either detected via newborn hearing screening or detected later in infancy. We analyzed the *GJB2* and *SLC26A4* genes for the presence of mutations, screened for the mitochondrial DNA (mtDNA) *A1555G* mutation, and screened for congenital CMV infection in DNA isolated from dried newborn blood spots. Results were obtained from 364 children. We established etiology for 60% of children. One or two known *GJB2* mutations were present in 82 children. Twenty-four children had one or two known *SLC26A4* mutations. *GJB2* or *SLC26A4* changes with unknown consequences on hearing were found in 32 children. The *A1555G* mutation was found in one child, and CMV infection was detected in 28 children. Auditory neuropathy spectrum disorder was confirmed in 26 children whose DNA evaluations were negative. A secondary objective was to investigate the relationship between etiology and audiological outcomes over the first 3 years of life. Regression analysis was used to investigate the relationship between hearing levels and etiology. Data analysis does not support the existence of differential effects of etiology on degree of hearing loss or on progressiveness of hearing loss.

## Introduction

Permanent childhood hearing loss, which occurs in 1–2 per 1000 live births [1], is associated with significant under-achievement in education and lifetime costs of care and lost productivity [2]. Universal newborn hearing screening (UNHS) has been adopted in many regions as a means to speed diagnosis and intervention. Despite its widespread implementation, the available evidence on whether earlier intervention improves long-term speech and language outcomes is flawed in important ways, including cohort composition and potential for systematic bias (reliance on parent-report measures only, non-blinded assessment, and selective follow up) [3]. To address the question of whether, at a population level, UNHS is effective in reducing the gap in speech and language skills between hearing-impaired children and their normal-hearing peers, we conducted the Longitudinal Outcomes of Children with Hearing Impairment (LOCHI) study to directly compare outcomes of children with or without access to UNHS in a prospective manner. The existence of uniform auditory intervention for all hearing-impaired children in Australia, within the government-funded national service provision system (Australian Hearing), serves to minimize variables that might affect outcomes.

Childhood hearing loss is an etiologically heterogeneous condition, caused by environmental factors and/or genetic factors. Despite this heterogeneity, mutations in the *GJB2* (connexin 26, Cx26) gene, the *SLC26A4* (pendred) gene and in the mitochondrial DNA at position 1555 (*A1555G* mutation) and congenital cytomegalovirus (CMV) infection have been considered as the major causes of congenital hearing loss in developed countries [4]. No previous population-based longitudinal studies on child outcomes have examined etiologies and related these to audiological outcomes in the same cohort. We have performed molecular testing for the four common causes to determine the frequency of occurrence in a community sample, and examined the relationship between etiology and audiological outcomes of children over their first 3 years of life. We hypothesized that there will be no difference in hearing level between children with known etiologies and children with none of the etiologies (Hypothesis 1). We also hypothesized that there will be no difference in progression of hearing loss over the first 3 years of life between children with known etiologies and children with none of the tested etiologies (Hypothesis 2).

## Subjects and Methods

### Ethics Statement

The study was approved by institutional ethics review boards (Royal Children’s Hospital HREC 28055 and Australian Hearing HREC 2008-3).

### Subjects

The LOCHI study commenced in 2005, with recruitment completed in 2007. All families with children born in Australia between 2002 and 2007 and who presented for hearing services below 3 years of age at Australian Hearing (AH) were invited to participate. Parents of children enrolled in the LOCHI study provided written, informed consent to participate. All participants were invited to give additional informed consent for molecular testing. Of the 451 eligible children, written consent was obtained for 387 children for molecular testing. There was no significant difference in hearing loss (average of 0.5, 1, 2 kHz) between the consent group and the non-consent group (p = 0.605). A total of 364 Guthrie card samples were obtained from the custodian authorities of health records. The sample comprised 280 whose hearing loss was detected via UNHS, 64 who did not have access to UNHS and 20 whose screening status was unknown.

### Audiologic Assessments

In Australia, the hearing of newborns is screened using automated auditory brainstem response (AABR). Infants who do not pass hearing screening are referred for diagnostic audiological assessment. These include tympanometry, middle ear muscle reflexes, otoacoustic emissions (OAEs), click-evoked auditory brainstem response (ABR) testing, and ABR or auditory steady-state responses (ASSR) testing using frequency-specific stimuli. Following diagnosis, children are referred to AH for assessment of hearing and provision of amplification, at no cost to families. Electrophysiological tests results are converted to estimated behavioral thresholds for fitting of hearing aids [5], [6]. Behavioral hearing thresholds are obtained using visual reinforcement audiometry or conditioned play audiometry, depending on the child’s age and ability. According to national protocols, hearing levels are monitored at regular intervals by experienced AH pediatric audiologists.

### Characteristics of Hearing Loss

The hearing thresholds of participants from inception to 44 months of age were retrieved from clinical records held at the AH national database, with written permission from parents. Hearing thresholds at 0.25, 0.5, 1, 2, and 4 kHz for each ear were obtained. These audiometric thresholds were used to characterize evolution of children’s hearing loss over the first 3 years of life as fluctuating, progressive, or stable. In accordance with the audiological protocol of AH, fluctuating hearing loss is defined as a change in hearing threshold of 15 dB or greater at any octave frequency between 0.5 and 4 kHz, but subsequently recover over the period of investigation. Progressive hearing loss is defined as a decrease in 10 dB or greater at two or more adjacent frequencies between 0.5 and 4 kHz or a decrease in 15 dB at one octave frequency in the same frequency range over the period of investigation. Longitudinal audiograms that do not demonstrate the changes specified above are labeled as stable.

ANSD is characterized by a dysfunction in neural/brainstem transmission of auditory stimuli in the presence of normal cochlear outer hair cell function. The clinical presentation is an absent or abnormal ABR responses and the presence of otoacoustic emissions or cochlear microphonics.

Information about enlarged vestibular aqueduct (EVA) was obtained from clinical records held at the AH national database. The diagnosis of EVA was based on CT scan. Radiographic imaging was not part of routine clinical service offered to children identified with hearing loss.

### Etiologic Evaluation

Lysates for molecular screening were prepared from Guthrie blood spots, using a modification of the method described by Barbi *et al*. [7]. Briefly, 3 mm diameter disks were punched from the Guthrie blood spot and soaked in 100 µl lysis buffer (50 mM Tris-HCl (pH 8.5); 50 mM NaCl; 2.5 mM MgCl_2_; 1 mg/ml Proteinase K; 1% Tween-20) at 56°C for 1 hour. Following incubation at 100°C for 7 mins. the samples were rapidly cooled on ice. The samples were centrifuged (10 minutes at 10,000 rpm) and the supernatants frozen for >4 hours at −80°C.

Lysates were screened for mutations in the coding region of *GJB2* (exon 2) and the splice site mutation in intron 1 (IVS1+1 G>A). Exon 2 PCR products were sequenced in both directions. The IVS1+1 G>A mutation was screened for by Hph1 digestion of a PCR product which spans the splice site. If a single mutation in *GJB2* was found, connexin-30 deletions del(GJB6-D13S1830) and del(GJB6d13s1854) were screened for as described by Castillo *et al.* [8].

Each of the *SLC26A4* gene exons was PCR amplified. Following nested PCR, the products were analyzed by High Resolution Melt (HRM) on the Corbett Rotorgene 6000 (Corbett Life Science, Australia). Primary PCR products were sequenced when HRM variants were identified.

We used *in silico* approaches to assess if unreported changes in the *GJB2* and *SLC26A4* genes are likely to affect protein function: PolyPhen-2 [9], SIFT BLink and SIFT Sequence [10].

The mitochondrial 12S rRNA *A1555G* mutation introduces a *HaeIII* restriction enzyme site. PCR products were digested with *Hae*III and analyzed by agarose gel electrophoresis.

The presence of CMV DNA was determined in a multiplex, quantitative, real-time PCR assay, using the CMV and CPOL probes published in Sanchez *et al*. [11] with the probes labelled as described in Scanga *et al*. [12]. Fifteen µl DNA lysate isolated from a disk was used in the PCR reaction, allowing two real-time PCR reactions to be analyzed from each 3 mm disk. The CMV tests were replicated on a second disk.

### Analysis

The results are summarized as means and percentages. To investigate the relationship between etiology and hearing thresholds at 3 years of age (Hypothesis 1), multiple regression analysis was carried out using the four-frequency average hearing loss (4FAHL, average of hearing threshold levels at 0.5, 1, 2, and 4 kHz in the better ear at 3 years of age) as dependent variable and etiology as a predictor variable. To determine the evolution of hearing loss, children were grouped according to the AH national protocols into those with fluctuating, progressive, or stable hearing loss. To consider the relation between progression of hearing loss and etiology (Hypothesis 2), a mixed-effects model was fitted using the longitudinal 4FA HL (four frequency average (average of 0.5, 1, 2, and 4 kHz) hearing level) data as dependent variable, the fixed effects were age (continuous variable), the seven etiologies (one mutation for *GJB2*, two mutations for *GJB2*, one mutation for *SLC26A4* gene, two mutations for *SLC26A4* gene, CMV infection, enlarged vestibular aqueduct (EVA), conductive loss) as two-category predictors, and the interactions between etiologies and age. We used two-tailed tests for all analyses and set statistical significance at p<0.05. The statistical analysis was performed using Statistica (Statsoft Inc, 2005) and R [13] with the additional packages ggplot2 [14] and nlme [15].

## Results

We use the wording “reported mutations” for DNA and protein changes classified as causative mutations in *Cx26* and *SLC26A4* mutation databases [16], [17], [18]. DNA changes with uncertain consequence on hearing are referred to as “ambiguous changes”.

**Figure 1:**
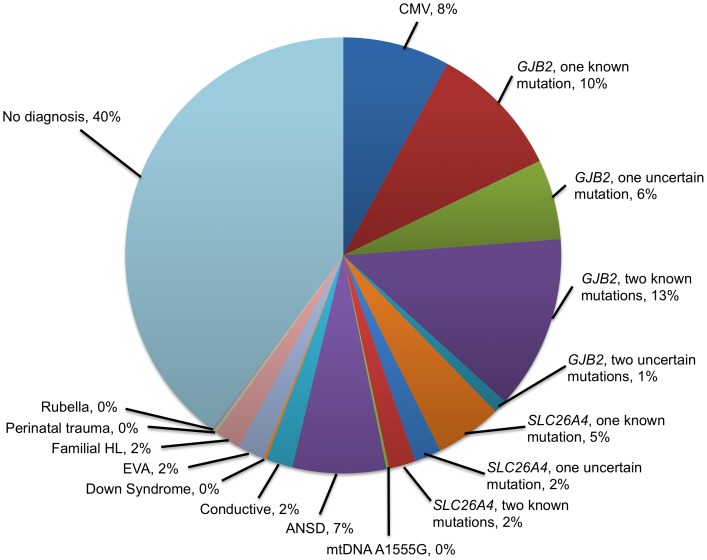
Results of the search for hearing loss etiology in 364 Australian children.

Figure 1 shows the distribution of predicted diagnoses. Reported mutations or congenital CMV infection were identified in 135 (37%) children, and ambiguous changes in the *GJB2* or *SLC26A4* gene were detected in 31 (9%) children. We did not detect mutations or ambiguous changes or evidence of congenital CMV infection in 198 (54%) children. These findings were supplemented by information retrieved from clinical records and parental reports. The cause of hearing loss remained unknown for 146 children (40%).

Reported mutations of *GJB2* were present in 82 children. Of these, 48 (58.5%) had two known mutations and 35 (41.5%) children had one mutation. The inheritance is in all cases compatible with a recessive inheritance pattern. Details of mutations and the evolution of hearing loss for individual children are shown in Table S1. Stable, fluctuating and progressive hearing losses are associated with GJB2 mutations, but specific genotypes do not appear to be exclusively associated with a phenotype. We assume that the hearing loss in children with two known mutations is attributable to *GJB2.* Of the children with two mutations of Cx26, one child passed hearing screening at birth, but was diagnosed with moderate hearing loss at 19.4 months of age. Another child was diagnosed with auditory neuropathy spectrum disorder (ANSD), and two children had EVA. Ambiguous changes in the *GJB2* gene were detected in 24 children, 22 of whom have one mutation and the remaining 2 have two mutations.

Reported mutations of *SLC26A4* [17], [18] were found in 24 children. Of these, seven (29.2%) had two known mutations, one of whom was also positive for CMV infection. Seventeen (70.8%) children carried one known *SLC26A4* mutation, two of whom also carried one uncertain mutation and four others also carried *GJB2* mutations. For 8 other children, one ambiguous change of the *SLC26A4* gene was detected. Details of mutations and evolution of hearing thresholds are shown in Table S2.

Mutations in the *SLC26A4* gene are associated with Pendred syndrome (PS, MIM 274600) and non-syndromic enlarged vestibular aqueduct (EVA, MIM 600791). Three children who carried one mutation of *SLC26A4* also had EVA, and one child who carried two mutations of *SLC26A4* also had EVA. EVA was also found for 4 children who had *Cx26* mutations, 1 who had CMV infection, and 8 who had normal results for the four causes tested. The number of children who had EVA could not be ascertained because information was available only for those with imaging results recorded in their clinical files.

The mtDNA *A1555G* mutation was detected in one child. The mutation appeared homoplasmic on agarose gel electrophoresis. The child has bilateral ANSD with profound hearing loss. It is not known whether the child had been exposed to aminoglycosides.

Cytomegalovirus infection was detected in 28 children. Of these, one child passed UNHS but was diagnosed with meningitis and hearing loss at 5.1 months of age. A second child with congenital CMV infection also had one mutation of Cx26, and was referred for unilateral hearing loss after screening. One CMV positive child also carried two mutations of the *SLC26A4* gene. Details of the evolution of hearing loss for children who are CMV positive are presented in Table S3.

In the regression analysis examining the relation between etiology and hearing threshold levels at 3 years of age, the overall p-level for the regression model was 0.27. Table 1 shows the estimate and 95% confidence interval for the regression coefficients. For each of the etiologies tested, there was insufficient evidence at the 5% significance level to reject the null hypothesis that the average hearing loss (4FA HL) for children with that etiology is the same as children without the etiology.

**Table 1:** Multiple regression analysis of hearing loss at 3 years of age with respect to etiology.

Predictor	Estimate	95% CI	p-value
Cx26, one mutation	−7.8	(−20.8, 5.1)	0.23
Cx26, two mutations	7.4	(−3.3, 18.1)	0.17
SLC26A4, one mutation	−7.0	(−21.0, 7.0)	0.32
SLC26A4, two mutations	−3.4	(−34.8, 27.9)	0.83
CMV	9.9	(−3.9, 23.8)	0.16
EVA	0.2	(−14.9, 15.3)	0.98
Conductive	−7.2	(−29.8, 15.3)	0.53

The estimated effect size, 95% confidence interval (CI), and the probability level (p-value) for each predictor term are shown.

Table 2 shows the estimates of the effect of etiology on progression of hearing loss over the first 3 years of life. For each of the seven etiologies investigated, there was insufficient evidence at the 5% significance level to reject the null hypothesis that the average rate of change of 4FA HL in the better ear for children with that etiology is the same as children without the etiology.

**Table 2:** Progression of hearing loss over the first 3 years of life, expressed in terms of slope in dB/year, with respect to predictors.

Predictor	Estimate	95% CI	p-value
Age	1.99	(−0.42, 4.41)	0.11
Cx26, one mutation×Age	−2.22	(−6.31, 1.87)	0.29
Cx26, two mutations×Age	1.12	(−2.38, 4.61)	0.53
SLC26A4, one mutation×Age	2.52	(−1.80, 6.84)	0.25
SLC26A4, two mutations×Age	0.18	(−8.11, 8.47)	0.97
CMV×Age	2.57	(−2.01, 7.15)	0.27
EVA×Age	3.97	(−0.64, 8.58)	0.09
Conductive×Age	−3.71	(−9.53, 2.11)	0.21

The estimated effect size, 95% confidence interval (CI), and the probability level (p-value) for each predictor term are shown.

## Discussion

The incidence of permanent childhood hearing loss is 1 to 1.2 per 1000 at birth increasing to about 1.5 by 3 years of age [19] in Australia where compliance with confirmatory testing after hearing screening is excellent and loss to follow-up for intervention is below 1%. As a sub-component of the LOCHI study, the present study conducted molecular testing of dried newborn blood spots for *GJB2*, *SLC26A4*, *A1555G* and CMV infection, and established a likely etiologic diagnosis for 166 (45.6%) of the 364 cases tested. Of the causes identified, a genetic cause was present in 138 children (83.1%) and an environmental cause in 28 children (16.9%).

It has previously been suggested that hearing loss attributable to congenital CMV may be progressive in >50% of cases [20]. However, we did not find this trend when examining hearing level data of children from diagnosis to 3 years of age. Whether the progression extends beyond this young age will be revealed in future follow-ups within the context of the LOCHI study. It is possible that some children with progressive hearing loss had congenital CMV infection that was not detected, as the use of Guthrie blood spot DNA in CMV testing may result in false negatives [21]. Dilution studies suggest that our CMV assay will detect the presence of a few copies of CMV DNA present in the 15 µl lysate used in the PCR reaction. Even with such sensitivity, CMV levels might be too low to be detected in Guthrie blood cards. More sensitive is the detection of CMV virus in the newborn’s blood, saliva urine or other tissues or body fluids within 2–3 weeks of birth. However, such tissues or fluids were not available in this cohort. Our finding that 8% of blood spots were positive for CMV is likely to be an underestimation of the true number of newborn Australian children with a congenital CMV infection. This frequency of infection is indeed lower than the 18.8% (9 of 48 infants with hearing loss) at a tertiary center of UNHS in Flanders [22]. In that study, CMV was detected through PCR assays with the blood spots on Guthrie cards, viral cultures, or blood serological tests. The present findings are nonetheless consistent with those reported by Stehel *et al* for infants at a hospital in the US showing that 6% (16 of 256 infants with hearing loss) were CMV positive through urine cultures [23]. Even though hearing loss in children with congenital CMV infection is likely to have been caused by the infection, we cannot conclude that this is the case in all children because most children with congenital CMV infection do not develop hearing loss [20].

Looking specifically at the genetic causes of hearing loss in our population (n = 138), mutations in the *GJB2* gene accounted for 76.1% of the genetic cases. This frequency of occurrence is higher than the 50% previously reported for congenital, autosomal recessive, nonsyndromic, sensorineural hearing loss in the white population [24]. Consistent with previous studies on children of northern European ancestry [22], [25], the c35delG (35delG mutation) variant was the most common allele variant of GJB2, with 60 of 135 mutant alleles (44%) being 35delG. As shown in Table S1, there was a variable genotype-phenotype correlation even among children homozygous for 35delG. Our finding that progressiveness of hearing loss in children with *GJB2* mutations was not significantly different from those without the mutations is consistent with previous reports [26], [27]. Unlike previous reports that revealed an association of biallellic truncating mutations (such as 35delG) with more severe loss than those with biallellic non-truncating mutations [28], our study did not reveal such a relation between mutations and hearing level.

One known mutation in the *GJB2* gene was detected in 35 children. The pathogenic effect of these single mutations are uncertain, but we have attributed the hearing losses to thise *GJB2* gene changes, because the proportion of children with hearing loss and one detectable recessive *GJB2* mutation is significantly higher than that of the general population (approximately 1%) [29]. The pathogenic effects of a number of other changes in the *GJB2* gene of 24 children are also ambiguous. We have classified the V27I and E114G changes, when together, as a mutation in accordance with the *Connexins and Deafness Homepage* [16]. However, recent data suggest that the V27I+E114G changes might not be pathogenic [30]. Although sometimes classified as polymorphisms [16], it is likely that M34T [28], [31], [32], [33], [34], R127H [35], [36], [37], M195V [38], [39], and I203T [40], [41] contribute to hearing loss. The V153I change is usually considered to be a polymorphism, although it has been suggested that the mutant *GJB2* protein is unable to form functional gap junction channels [42]. We have previously reported the T186A change [43], but the pathogenicity of this change has not been established. The T186A change was seen in two affected siblings together with the 35delG mutation. PolyPhen-2, SIFT BLink and SIFT Sequence analyses all predict this change to affect protein function (Table S4). The K108N change has not been reported before, but the *in silico* analysis suggests that is unlikely to cause hearing loss. Although SIFT Sequence analysis predicts that it is “likely to affect protein function”, PolyPhen-2 and SIFT BLink predict it is benign (Table S4).

Previous studies have shown the presence of two *SLC26A4* mutations in many patients with Pendred syndrome, whereas one mutation is common in patients with EVA [44], [45] *SLC26A4* mutations [17], [18] were found in 32 children. Of these, 7 (21.9%) had two known mutations, 17 (53.1%) had one known mutation and 8 (25.0%) had one uncertain mutation. As shown in Table S2, there was a complex genotype-phenotype correlation. The c.919_936delinsCCCCA change has not been described before, but the exon 8 mutation results in a frameshift and premature termination of the protein. As with the *GJB2* mutations, the pathogenic effects of some of the *SLC26A4* changes haven’t been fully established and are controversial. This is the case for L597S [45], [46], [47], [48], [49], [50], [51], [52], IVS1-2A>G [51], and F335L [45]. PolyPhen-2 and SIFT Sequence predict the L597S and F335L changes are “probably damaging”, while SIFT BLink suggests they affect protein function (Table S4). Only 4 of the cases identified with one or two mutations in the SLC26A4 also had EVA. The status for the remaining cases could not be ascertained from clinical records. Therefore, the pathogenicity of detected mutations in those with one mutation is uncertain.

We detected 3 previously unreported missense changes: R43H, R79Q and S780F. A mutation, R43S, has been reported in R43 suggesting that this amino acid might be important for protein function [17]. R43 and R79 are both highly conserved amino acids suggesting that the R43H and R79Q changes might affect SLC26A4 function. However, PolyPhen-2, SIFT BLink and SIFT Sequence analyses predict the R43H change to be benign. PolyPhen-2 predicts the R79Q change to be “probably damaging” to protein function, while the two SIFT programs predict that the change is “tolerated” (Table S4). Despite being the C-terminal amino acid, S780 is conserved in most species. However, in the Common Shrew (Sorex araneus) the corresponding amino acid is a phenylalanine. This suggests that the S780F change might be a polymorphism. PolyPhen-2 predicts this change to be benign, while SIFT BLink and SIFT Sequence predict that the change affects protein function (Table S4). However, the *in silico* predictions at the C-terminal amino acid are with low confidence because only short sequence regions can be compared.

To assess the contribution of single mutations in the *GJB2* or *SLC2A4* gene to a hearing loss is challenging. The study by Wang et al [53] in which 14,913 newborn Chinese children had both a newborn hearing test and a genetic screen for *GJB2* and *SLC2A4* mutations showed significantly increased risks of hearing loss in *GJB2* and *SLC2A4* heterozygous carrier children.

The *A1555G* mtDNA mutation increases susceptibility to aminoglycoside induced hearing loss. Because many newborns and young children are exposed to aminoglycoside antibiotics, its potential presence is often of concern to health professionals and families. The *A1555G* mtDNA mutation was found in only one of the 364 hearing-impaired children. Our result is consistent with previous observations that this mutation is not a common cause of hearing loss in children less than 3 years of age in the general Australian population. [43].

A diagnosis of ANSD was established for 39 children. Of the children diagnosed with ANSD, four had one *SLC26A4* mutation also; six had one GJB2 mutation and one child had two GJB2 mutations. Twenty-six of the children with ANSD did not have detectable mutations or evidence of CMV infection. The presence of ANSD results in degraded oral language development.

The limitations of this study include its reliance on newborn blood spots for CMV testing. Supplementing UNHS with molecular testing would enable pre-symptomatic detection of preventable hearing loss for some children. Despite testing for four common causes of childhood hearing loss, the etiology for 54% of the cohort remains unknown. Future molecular testing for etiology of hearing loss will need to include mutations in other genes, such as *OTOF*, encoding otoferlin, in which mutations are commonly found in children with ANSD. It was not possible to do a comprehensive audiological and genetic study of parents, siblings and other relatives of affected children. Such study could be helpful in assessing the inheritance and consequence of a *GJB2* or *SLC26A4* change. Future follow-up of the present cohort as part of the longitudinal study will examine the relation between an identified genetic/viral cause and the type and degree of hearing loss as the children grow.

The strengths of the LOCHI study include its longitudinal, community-based design, with prospective, repeated measurements of hearing and developmental outcomes. The longer term audiological outcomes of children with different etiologies will be evaluated.

### Conclusion

This study presents the first comprehensive evaluation of four common causes of childhood hearing loss in a community cohort. After etiologic evaluation of 364 children identified with hearing loss below 3 years of age, a genetic diagnosis could be made in 46% of those children. Approximately another 14% of children had clinical abnormalities or syndromes without identified *GJB2* or *SLC26A4* mutations. Our findings provide a basis for future studies to determine the impact of mutations of the *GJB2* and *SLC26A4* genes and congenital CMV infection on speech and language outcomes in children with hearing loss. Establishing the etiology of an identified hearing loss provides answers to parents, facilitates planning of hearing loss management and provides information that is relevant to the prognosis for the child.

## Supporting Information

Table S1 **Genotype-phenotype correlation in children with GJB2 mutations and evolution of hearing loss.**
(DOC)

Table S2 **Genotype-phenotype correlation in children with** ***SLC26A4*** **mutations and evolution of hearing loss.** Children with *SLC26A4* mutations with additional diagnostic information regarding enlarged vestibular aqueduct (EVA), auditory neuropathy spectrum disorder (ANSD) and congenital cytomegalovirus infection (CMV) are also indicated.
(DOC)

Table S3 **Hearing thresholds at time of diagnosis and evolution in children with hearing loss attributable to congenital CMV infection.**
(DOC)

Table S4 **PolyPhen-2, SIFT BLink and SIFT Sequence predictions of novel or controversial amino acid changes on** ***GJB2*** **and** ***SLC26A4*** **gene product functions.**
(DOCX)

## References

[1] Davis A, Davis KAS (2011) Chapter 5. Descriptive epidemiology of childhood hearing impairment. In: , editor. Comprehensive Handbook of Pediatric Audiology: Plural Publishing, Abingdon, UK. pp. 85–111.

[2] Access Economics (2006) Listen Hear. The Economic Impact and Cost of Hearing Loss in Australia.

[3] Thompson DC, McPhillips H, Davis RL, Lieu TL, Homer CJ, et al (2001) Universal newborn hearing screening: summary of evidence. JAMA 286: 2000–2010. 11667937 10.1001/jama.286.16.2000

[4] Morton CC, Nance WE (2006) Newborn hearing screening–a silent revolution. N Engl J Med 354: 2151–2164. 16707752 10.1056/NEJMra050700

[5] King AC, Parkinson KN, Adamson AJ, Murray L, Besson H, et al (2011) Correlates of objectively measured physical activity and sedentary behaviour in English children. Eur J Public Health 21: 424–431. 20650946 10.1093/eurpub/ckq104

[6] Vander Werff KR, Prieve BA, Georgantas LM (2009) Infant air and bone conduction tone burst auditory brain stem responses for classification of hearing loss and the relationship to behavioral thresholds. Ear Hear 30: 350–368. 19322084 10.1097/AUD.0b013e31819f3145

[7] Barbi M, Binda S, Primache V, Caroppo S, Dido P, et al (2000) Cytomegalovirus DNA detection in Guthrie cards: a powerful tool for diagnosing congenital infection. J Clin Virol 17: 159–165. 10996112 10.1016/s1386-6532(00)00089-5

[8] del Castillo FJ, Rodriguez-Ballesteros M, Alvarez A, Hutchin T, Leonardi E, et al (2005) A novel deletion involving the connexin-30 gene, del(GJB6-d13s1854), found in trans with mutations in the GJB2 gene (connexin-26) in subjects with DFNB1 non-syndromic hearing impairment. J Med Genet 42: 588–594. 15994881 10.1136/jmg.2004.028324 PMC1736094

[9] Adzhubei IA, Schmidt S, Peshkin L, Ramensky VE, Gerasimova A, et al (2010) A method and server for predicting damaging missense mutations. Nature methods 7: 248–249. 20354512 10.1038/nmeth0410-248 PMC2855889

[10] Kumar P, Henikoff S, Ng PC (2009) Predicting the effects of coding non-synonymous variants on protein function using the SIFT algorithm. Nature protocols 4: 1073–1081. 19561590 10.1038/nprot.2009.86

[11] Sanchez JL, Storch GA (2002) Multiplex, quantitative, real-time PCR assay for cytomegalovirus and human DNA. J Clin Microbiol 40: 2381–2386. 12089251 10.1128/JCM.40.7.2381-2386.2002 PMC120584

[12] Scanga L, Chaing S, Powell C, Aylsworth AS, Harrell LJ, et al (2006) Diagnosis of human congenital cytomegalovirus infection by amplification of viral DNA from dried blood spots on perinatal cards. J Mol Diagn 8: 240–245. 16645211 10.2353/jmoldx.2006.050075 PMC1867599

[13] The “R Project for Statistical Computing” website. R Development Core Team. A language and environment for statistical computing (Version 2.13.1). Available: http://www.r-project.org/. Accessed 2012 May 8.

[14] The “ggplot2: An implementation of the Grammar of Graphics” website. Wickham H and Chang W. Available: http://cran.r-project.org/package=ggplot2. Accessed 2012 May 8.

[15] The “nlme: Linear and Nonlinear Mixed Effects Models” website. Pinheiro J, Bates, D., DebRoy, S., Sarkar, D. & R Development Core Team. Available: http://cran.r-project.org/package=nlme. Accessed 2012 May 8.

[16] Connexin-deafness Homepage website. Ballana E, Ventayol M, Rabionet R, Gasparini P, Estivill X. Available: http://davinci.crg.es/deafness/. Accessed 2012 Aug 26.

[17] Pendred/BOR Homepage website. Chang E, Kölln KA, Nishimura CJ, Fischer S, Smith RJH. Available: http://www.healthcare.uiowa.edu/labs/pendredandbor/. Accessed 2012 Aug 26.

[18] Deafness Gene Mutation Database website. Harvard Medical School Center for Hereditary Deafness (2012). Available: http://hearing.harvard.edu/genepages/pdsdream.htm. Accessed 2012 Aug 26.

[19] Australian Hearing website Demographic Details of Persons under the age of 21 years with a Hearing Impairment who are fitted with a Hearing Aid - 2011. Available: http://www.hearing.com.au/digitalAssets/13809_1352761241226_Demographics%20of%20Persons%20under%20the%20age%20of%2021%20years%20with%20Hearing%20Aids.pdf. Accessed 2012 May 8.

[20] Fowler KB, Boppana SB (2006) Congenital cytomegalovirus (CMV) infection and hearing deficit. J Clin Virol 35: 226–231. 16386462 10.1016/j.jcv.2005.09.016

[21] Boppana SB, Ross SA, Novak Z, Shimamura M, Tolan RW Jr, et al (2010) Dried blood spot real-time polymerase chain reaction assays to screen newborns for congenital cytomegalovirus infection. Jama 303: 1375–1382. 20388893 10.1001/jama.2010.423 PMC2997517

[22] Declau F, Boudewyns A, Van den Ende J, Peeters A, van den Heyning P (2008) Etiologic and audiologic evaluations after universal neonatal hearing screening: analysis of 170 referred neonates. Pediatrics 121: 1119–1126. 18519481 10.1542/peds.2007-1479

[23] Stehel EK, Shoup AG, Owen KE, Jackson GL, Sendelbach DM, et al (2008) Newborn hearing screening and detection of congenital cytomegalovirus infection. Pediatrics 121: 970–975. 18450901 10.1542/peds.2006-3441

[24] Smith RJ, Bale JF Jr, White KR (2005) Sensorineural hearing loss in children. Lancet 365: 879–890. 15752533 10.1016/S0140-6736(05)71047-3

[25] Denoyelle F, Weil D, Maw MA, Wilcox SA, Lench NJ, et al (1997) Prelingual deafness: high prevalence of a 30delG mutation in the connexin 26 gene. Hum Mol Genet 6: 2173–2177. 9336442 10.1093/hmg/6.12.2173

[26] Denoyelle F, Marlin S, Weil D, Moatti L, Chauvin P, et al (1999) Clinical features of the prevalent form of childhood deafness, DFNB1, due to a connexin-26 gene defect: implications for genetic counselling. Lancet 353: 1298–1303. 10218527 10.1016/S0140-6736(98)11071-1

[27] Murgia A, Orzan E, Polli R, Martella M, Vinanzi C, et al (1999) Cx26 deafness: mutation analysis and clinical variability. J Med Genet 36: 829–832. 10544226 PMC1734250

[28] Snoeckx RL, Huygen PL, Feldmann D, Marlin S, Denoyelle F, et al (2005) GJB2 mutations and degree of hearing loss: a multicenter study. Am J Hum Genet 77: 945–957. 16380907 10.1086/497996 PMC1285178

[29] Dahl HH, Saunders K, Kelly TM, Osborn AH, Wilcox S, et al (2001) Prevalence and nature of connexin 26 mutations in children with non-syndromic deafness. Med J Aust 175: 191–194. 11587277 10.5694/j.1326-5377.2001.tb143093.x

[30] Tekin M, Xia XJ, Erdenetungalag R, Cengiz FB, White TW, et al (2010) GJB2 mutations in Mongolia: complex alleles, low frequency, and reduced fitness of the deaf. Ann Hum Genet 74: 155–164. 20201936 10.1111/j.1469-1809.2010.00564.x PMC4739516

[31] Cucci RA, Prasad S, Kelley PM, Green GE, Storm K, et al (2000) The M34T allele variant of connexin 26. Genet Test 4: 335–344. 11216656 10.1089/109065700750065063

[32] Houseman MJ, Ellis LA, Pagnamenta A, Di WL, Rickard S, et al (2001) Genetic analysis of the connexin-26 M34T variant: identification of genotype M34T/M34T segregating with mild-moderate non-syndromic sensorineural hearing loss. J Med Genet 38: 20–25. 11134236 10.1136/jmg.38.1.20 PMC1734724

[33] Kenna MA, Feldman HA, Neault MW, Frangulov A, Wu BL, et al (2010) Audiologic phenotype and progression in GJB2 (Connexin 26) hearing loss. Arch Otolaryngol Head Neck Surg 136: 81–87. 20083784 10.1001/archoto.2009.202 PMC4528189

[34] Teek R, Kruustuk K, Zordania R, Joost K, Reimand T, et al (2010) Prevalence of c.35delG and p.M34T mutations in the GJB2 gene in Estonia. Int J Pediatr Otorhinolaryngol 74: 1007–1012. 20708129 10.1016/j.ijporl.2010.05.026

[35] Dahl HH, Tobin SE, Poulakis Z, Rickards FW, Xu X, et al (2006) The contribution of GJB2 mutations to slight or mild hearing loss in Australian elementary school children. J Med Genet 43: 850–855. 16840571 10.1136/jmg.2006.042051 PMC2563186

[36] Palmada M, Schmalisch K, Bohmer C, Schug N, Pfister M, et al (2006) Loss of function mutations of the GJB2 gene detected in patients with DFNB1-associated hearing impairment. Neurobiol Dis 22: 112–118. 16300957 10.1016/j.nbd.2005.10.005

[37] Thonnissen E, Rabionet R, Arbones ML, Estivill X, Willecke K, et al (2002) Human connexin26 (GJB2) deafness mutations affect the function of gap junction channels at different levels of protein expression. Hum Genet 111: 190–197. 12189493 10.1007/s00439-002-0750-2

[38] Dai P, Yu F, Han B, Liu X, Wang G, et al (2009) GJB2 mutation spectrum in 2,063 Chinese patients with nonsyndromic hearing impairment. J Transl Med 7: 26. 19366456 10.1186/1479-5876-7-26 PMC2679712

[39] Tsukada K, Nishio S, Usami S (2010) A large cohort study of GJB2 mutations in Japanese hearing loss patients. Clin Genet 78: 464–470. 20497192 10.1111/j.1399-0004.2010.01407.x

[40] Han SH, Park HJ, Kang EJ, Ryu JS, Lee A, et al (2008) Carrier frequency of GJB2 (connexin-26) mutations causing inherited deafness in the Korean population. J Hum Genet 53: 1022–1028. 19043807 10.1007/s10038-008-0342-7

[41] Kim HJ, Park CH, Lee KO, Won HH, Ko MH, et al (2010) Sequence Variations and Haplotypes of the GJB2 Gene Revealed by Resequencing of 192 Chromosomes from the General Population in Korea. Clin Exp Otorhinolaryngol 3: 65–69. 20607074 10.3342/ceo.2010.3.2.65 PMC2896735

[42] Mese G, Londin E, Mui R, Brink PR, White TW (2004) Altered gating properties of functional Cx26 mutants associated with recessive non-syndromic hearing loss. Hum Genet 115: 191–199. 15241677 10.1007/s00439-004-1142-6

[43] Siemering K, Manji SS, Hutchison WM, Du Sart D, Phelan D, et al.. (2006) Detection of mutations in genes associated with hearing loss using a microarray-based approach. J Mol Diagn 8: 483–489; quiz 528. 10.2353/jmoldx.2006.050147 PMC1867613 16931589

[44] Azaiez H, Yang T, Prasad S, Sorensen JL, Nishimura CJ, et al (2007) Genotype-phenotype correlations for SLC26A4-related deafness. Hum Genet 122: 451–457. 17690912 10.1007/s00439-007-0415-2 PMC10519375

[45] Choi BY, Stewart AK, Madeo AC, Pryor SP, Lenhard S, et al (2009) Hypo-functional SLC26A4 variants associated with nonsyndromic hearing loss and enlargement of the vestibular aqueduct: genotype-phenotype correlation or coincidental polymorphisms? Hum Mutat 30: 599–608. 19204907 10.1002/humu.20884 PMC2663020

[46] Albert S, Blons H, Jonard L, Feldmann D, Chauvin P, et al (2006) SLC26A4 gene is frequently involved in nonsyndromic hearing impairment with enlarged vestibular aqueduct in Caucasian populations. Eur J Hum Genet 14: 773–779. 16570074 10.1038/sj.ejhg.5201611

[47] Blons H, Feldmann D, Duval V, Messaz O, Denoyelle F, et al (2004) Screening of SLC26A4 (PDS) gene in Pendred’s syndrome: a large spectrum of mutations in France and phenotypic heterogeneity. Clin Genet 66: 333–340. 15355436 10.1111/j.1399-0004.2004.00296.x

[48] Campbell C, Cucci RA, Prasad S, Green GE, Edeal JB, et al (2001) Pendred syndrome, DFNB4, and PDS/SLC26A4 identification of eight novel mutations and possible genotype-phenotype correlations. Hum Mutat 17: 403–411. 11317356 10.1002/humu.1116

[49] Fugazzola L, Cerutti N, Mannavola D, Crino A, Cassio A, et al (2002) Differential diagnosis between Pendred and pseudo-Pendred syndromes: clinical, radiologic, and molecular studies. Pediatr Res 51: 479–484. 11919333 10.1203/00006450-200204000-00013

[50] Pera A, Dossena S, Rodighiero S, Gandia M, Botta G, et al (2008) Functional assessment of allelic variants in the SLC26A4 gene involved in Pendred syndrome and nonsyndromic EVA. Proc Natl Acad Sci U S A 105: 18608–18613. 19017801 10.1073/pnas.0805831105 PMC2584577

[51] Pryor SP, Madeo AC, Reynolds JC, Sarlis NJ, Arnos KS, et al (2005) SLC26A4/PDS genotype-phenotype correlation in hearing loss with enlargement of the vestibular aqueduct (EVA): evidence that Pendred syndrome and non-syndromic EVA are distinct clinical and genetic entities. J Med Genet 42: 159–165. 15689455 10.1136/jmg.2004.024208 PMC1735974

[52] Yang T, Vidarsson H, Rodrigo-Blomqvist S, Rosengren SS, Enerback S, et al (2007) Transcriptional Control of SLC26A4 Is Involved in Pendred Syndrome and Nonsyndromic Enlargement of Vestibular Aqueduct (DFNB4). Am J Hum Genet 80: 1055–1063. 17503324 10.1086/518314 PMC1867094

[53] Wang QJ, Zhao YL, Rao SQ, Guo YF, He Y, et al (2011) Newborn hearing concurrent gene screening can improve care for hearing loss: a study on 14,913 Chinese newborns. Int J Pediatr Otorhinolaryngol 75: 535–542. 21329993 10.1016/j.ijporl.2011.01.016

