# Supplementary material for: Etiology and Audiological Outcomes at 3 Years for 364 Children in Australia
Source: PLoS One. 2013 Mar 28;8(3):e59624. doi: 10.1371/journal.pone.0059624 (PMC3610796; doi:10.1371/journal.pone.0059624)
Supplement: Table S1 — Genotype-phenotype correlation in children with GJB2 mutations and evolution of hearing loss. (DOC) [file pone.0059624.s001.doc]

**Supplementary Table S1.** Genotype-phenotype correlation in children with GJB2 mutations and evolution of hearing loss

|  |  |  | Diagnosis |  | 3 years of age |  | Evolution |
| --- | --- | --- | --- | --- | --- | --- | --- |
|  | *GJB2* | Other | HTL -left | HTL - right | HTL -left | HTL - right |  |
|  | **One mutation** |  |  |  |  |  |  |
| S6 | 269insT/N |  | 76.25 | 74 | 103.75 | 87.5 | Fluctuating/  Progressive |
| S26 | V37I/N |  | 93.75 | 93.75 | >100 | 115 | Stable |
| S53 | 35delG/N |  | 101.25 | 101.25 | 112.5 | >100 | Stable |
| S57 | V37I/N |  | 50 | 50 | 48.75 | 46.25 | Stable |
| S64 | I203T/N |  | 58.75 | 62.5 | 81.25 | 81.25 | Progressive |
| S87 | M34T/N |  | 62.5 | 62.5 | 70 | 70 | Stable |
| S90 | M34T/N |  | 37.5 | 37.5 | 38.75 | 38.75 | Stable |
| S94 | V27I+E114G/N |  | 36.25 | 36.25 | 57.5 | 47.5 | Progressive |
| S107 | Q124X/N |  | 42.5 | 42.5 | 27.5 | 32.5 | Improved |
| S109 | 35delG/N | ANSD  Cerebral Palsy | 75 | 75 | 52.5 | 52.5 | Stable |
| S123 | K108N /N | EVA  CMV | 32.5 | 41.25 | 45 | 53.75 | Fluctuating/  Progressive |
| S129 | R127H/N | ANSD | 52.5 | 52.5 | 58.75 | 58.75 | Stable |
| S136 | M195V/N |  | 30 | 95 | 23.75 | 82.5 | Stable |
| S142 | V37I/N | CMV | 71.25 | 30 | 91.25 | 38.75 | Progressive |
| S143 | I203T/N | ANSD | 62 | 63 | 72.5 | 47.5 | Stable |
| S149 | M34T/N | EVA | 20 | 60 | 28.75 | 58.75 | Stable |
| S204 | V153I/N |  | 70 | 70 | 70 | 68.75 | Stable |
| S232 | M34T/N | Meningitis | 87.5 | 87.5 | >100 | >100 | Stable |
| S237 | V27I+E114G/N |  | 53.5 | 56.65 | 62.5 | 61.25 | Stable |
| S256 | M34T/N | ANSD | 100 | 100 | >100 | >100 | Stable |
| S274 | 269insT/N | Other Syndrome | 111.25 | 115 | >100 | 107.5 | Stable |
| S321 | 167delT/N | EVA | 82.5 | 78.75 | 73.75 | 71.25 | Stable |
| S329 | M34T/N |  | 58.75 | 58.75 | 57.5 | 57.5 | Fluctuating |
| S337 | P70A/N |  | 38.75 | 38.75 | 45 | 47.5 | Stable |
| S348 | V27I+E114G/N | *SLC26A4*:  R776S/N | 15 | 16.25 | 17.5 | 17.5 | Stable |
| S355 | V27I+E114G/N | Conductive  Other Syndrome | 60 | 60 | 61.25 | 68.75 | Stable |
| S377 | M34T/N |  | 87.5 | 107.5 | >100 | 120 | Stable |
| S388 | E47X/N |  | 103.75 | 103.75 | >100 | >100 | Stable |
| S424 | V27I+E114G/N |  | 100 | 100 | >100 | >100 | Stable |
| S430 | 35delG/N |  | 41.25 | 47.5 | 58.75 | 58.75 | Stable |
| S462 | M34T/N | CMV  EVA | >90 | >90 | 120 | >100 | Stable |
| S464 | 35delG/N |  | 65 | 46.25 | 66.25 | 55 | Stable |
| S490 | R127H/N |  | 71.25 | 71.25 | 71.25 | 71.25 | Stable |
| S491 | M34T/N |  | 45 | 45 | 45 | 45 | Stable |
| S507 | IVS1+1 G>A/N |  | 93.75 | 80 | 65 | 65 | Fluctuating |
| S508 | M34T/N | *SLC26A4*:  IVS6 + 2T>C/N | 72.5 | 72.5 | 87.5 | 81.25 | Progressive |
| S513 | D13S1830/N |  | 40 | 40 | 41.25 | 41.25 | Fluctuating |
| S520 | M34T/N |  | 42.5 | 47.5 | 40 | 38.75 | Stable |
| S530 | V153I/N | ANSD | 70 | 70 | 52.5 | 52.5 | Fluctuating |
| S532 | M34T/N | ANSD | 36.25 | 36.25 | 37.5 | 50 | Stable |
| S606 | 290-291insA/N |  | 42.5 | 42.5 | 37.5 | 38.75 | Stable |
| S712 | V37I/N |  | 76.25 | 76.25 | 65 | 55 | Stable |
| S718 | 35delG/N |  | 80 | 80 | 78.75 | >100 | Stable |
| S813 | R127H/N |  | 65 | 45 | 37.5 | 50 | Fluctuating |
|  | **Two mutations** |  |  |  |  |  |  |
| S4 | 35delG/35delG |  | 86.25 | 86.25 | >100 | >100 | Stable |
| S19 | 35delG/35delG |  | 73.0 | 56.75 | 108.75 | 76.25 | Fluctuating |
| S37 | W24X/W24X |  | >90 | >90 | >100 | >100 | Stable |
| S40 | 235delC/V37I |  | 22.5 | 22.5 | 25 | 25 | Stable |
| S46 | 35delG/35delG |  | 53.75 | 53.75 | 58.75 | 52.5 | Stable |
| S48 | 35delG/35delG |  | 53.75 | 48.75 | 83.75 | 80 | Progressive |
| S54 | 35delG/35delG | *SLC26A4*:  T416P/N | 81.25 | 81.25 | >100 | >100 | Stable |
| S61 | 35delG/M34T |  | 55 | 50 | 85 | >100 | Progressive |
| S67 | 35delG/T186A |  | 40 | 43.75 | 37.5 | 46.25 | Stable |
| S76 | 167delT/235delC |  | 101.25 | 101.25 | >100 | >100 | Stable |
| S80 | D13S1830/D13S1830 |  | >90 | >90 | >100 | >100 | Stable |
| S85 | D13S1830/M34T |  | 45 | 45 | 56.25 | 56.25 | Stable |
| S86 | M34T/M34T | ANSD | 40 | 40 | 42.5 | 42.5 | Stable |
| S96 | V37I/V37I | EVA | 46.25 | 46.25 | 58.75 | 61.25 | Progressive |
| S105 | delE120/35delG |  | 33.75 | 33.75 | 46.25 | 46.25 | Fluctuating |
| S106 | delE120/35delG | EVA | 50 | 62.5 | 78.75 | >100 | Fluctuating/Progressive |
| S108 | V37I/299-300delAT |  | 46.25 | 45 | 46.25 | 46.25 | Stable |
| S124 | 35delG/D13S1830 |  | 80 | 75 | >100 | >100 | Stable |
| S127 | M1V/35delG | EVA | 53.75 | 53.75 | 81.25 | 81.25 | Fluctuating/Progressive |
| S130 | W24X/W24X |  | 100 | 100 | >100 | >100 | Stable |
| S153 | 35delG/V27I+E114G |  | 21.25 | 21.25 | 21.25 | 21.25 | Stable |
| S155 | 35delG/35delG |  | 40 | 47.5 | 47.5 | 47.5 | Stable |
| S156 | 35delG/M34T |  | 45.25 | 45.25 | 61.25 | 71.25 | Fluctuating |
| S208 | 35delG/I82M |  | 80 | 80 | 88.75 | 88.75 | Stable |
| S223 | 35delG/M34T |  | 45 | 50 | 52.5 | 56.25 | Stable |
| S224 | 35delG/W77R |  | 78.75 | 78.75 | 87.5 | >100 | Stable |
| S233 | 235delC/299-300delAT |  | 93.75 | 93.75 | >100 | 111.25 | Stable |
| S234 | 35delG/M34T |  | 36.25 | 36.25 | 27.5 | 25 | Fluctuating |
| S261 | V37I/V37I | *SLC26A4*:  D661E/N | 41.25 | 36.25 | 41.25 | 32.5 | Stable |
| S262 | 35delG/M34T |  | 52.5 | 62.5 | 55 | 57.5 | Stable |
| S301 | 35delG/35delG |  | 105 | 105 | 113.75 | >100 | Stable |
| S310 | 35delG/M34T |  | 33.75 | 62.5 | 45 | 45 | Fluctuating |
| S318 | V37I/V37I |  | 80 | 80 | 78.75 | 77.5 | Stable |
| S344 | T8M/V153I |  | 55 | 55 | 48.75 | 55 | Stable |
| S350 | 35delG/L90P |  | 83.75 | 83.75 | >100 | >100 | Stable |
| S370 | V37I/V84L |  | 40 | 40 | 60 | 58.75 | Progressive |
| S379 | 35delG/167delT |  | 117.5 | 110 | >100 | >100 | Stable |
| S386 | 35delG/35delG |  | 86.25 | 120 | 70 | >100 | Stable |
| S417 | 35delG/W77R |  | 90 | 90 | >100 | 107.5 | Stable |
| S444 | IVS1+1 G>A/IVS1+1 G>A |  | >90 | >90 | >100 | 106.3 | Stable |
| S460 | 35delG/35delG |  | >90 | >90 | 110 | >100 | Stable |
| S470 | L90P/IVS1+1 G>A |  | 47.5 | 47.5 | 42.5 | 70 | Fluctuating |
| S476 | 35delG/T186A | EVA | 57.5 | 52.5 | 63.75 | 57.5 | Stable |
| S477 | 35insG/299-300delAT |  | 83.25 | 94.25 | 87.5 | >100 | Fluctuating |
| S478 | 35delG/269insT | EVA | 55 | 60 | 61.25 | 63.75 | Stable |
| S486 | M34T/M34T |  | 31.25 | 52.5 | 25 | 28.75 | Fluctuating |
| S519 | 35delG/35delG |  | 75 | 75 | 100 | 65 | Progressive |
| S535 | 35delG/269insT |  | 45 | 45 | 53.75 | 53.75 | Progressive |
| S538 | 35delG/W24X |  | 62.5 | 63.75 | >100 | >100 | Fluctuating |
| S617 | V37I/V37I |  | 41.25 | 41.25 | 41.25 | 41.25 | Stable |
| S620 | V37I/V37I |  | 62.5 | 53.75 | 46.25 | 42.5 | Stable |
| S625 | T8M/V153I | SLC26A4:L597S/N  EVA | 43.75 | 78.75 | 51.25 | 80 | Fluctuating |
| S704 | 35delG/35delG |  | >90 | >90 | 113.75 | >100 | Stable |
| S706 | 35delG/D13S1830 |  | >90 | >90 | 107.5 | >100 | Stable |
| S716 | 35delG/R143W |  | 95 | 95 | 106.25 | >100 | Stable |
| S724 | 35delG/35delG |  | 58.75 | 58.75 | >100 | 82.5 | Progressive |
| S725 | 35delG/35delG |  | 68.75 | 68.75 | 66.25 | 66.25 | Stable |
| S806 | V37I/V37I |  | 35 | 35 | 41.25 | 37.5 | Stable |
| S815 | V37I/V37I |  | 51.25 | 37.5 | 50 | 31.25 | Stable |
| S823 | 35delG/35delG |  | 70 | 70 | 77.5 | 70 | Stable |
| S831 | 35delG/35delG |  | 105 | 105 | >100 | >100 | Stable |
| S861 | IVS1+1 G>A/M34T |  |  |  |  |  |  |
| S862 | 35delG/35delG |  | 95 | 95 | >100 | >100 | Stable |
