# Supplementary material for: Etiology and Audiological Outcomes at 3 Years for 364 Children in Australia
Source: PLoS One. 2013 Mar 28;8(3):e59624. doi: 10.1371/journal.pone.0059624 (PMC3610796; doi:10.1371/journal.pone.0059624)
Supplement: Table S2 — Genotype-phenotype correlation in children with SLC26A4 mutations and evolution of hearing loss. Children with SLC26A4 mutations with additional diagnostic information regarding enlarged vestibular aqueduct (EVA), auditory neuropathy spectrum disorder (ANSD) and congenital cytomegalovirus infection (CMV) are also indicated. (DOC) [file pone.0059624.s002.doc]

**Supplementary** **Table S2.** Genotype-phenotype correlation in children with *SLC26A4* mutations and evolution of hearing loss. Children with *SLC26A4* mutations with additional diagnostic information regarding enlarged vestibular aqueduct (EVA), auditory neuropathy spectrum disorder (ANSD) and congenital cytomegalovirus infection (CMV) are also indicated.

|  |  |  | Diagnosis | | 3 years of age | | Evolution |
| --- | --- | --- | --- | --- | --- | --- | --- |
| Participant | *SLC26A4* | Other | HTL -left | HTL - right | HTL -left | HTL - right |  |
|  | **One mutation** |  |  |  |  |  |  |
| S11 | R43H/N |  | 37.5 | 31.3 | 38.8 | 38.8 | Stable |
| S20 | R776S/N | ANSD | 51.7 | 53 | 58.8 | 61.3 | Stable |
| S30 | I455F/N |  | 52.0 | 51.5 | 66.3 | 58.8 | Progressive/ Fluctuating |
| S38 | F354S/N | ANSD | 70 | 87.5 | 35.0 | 35.0 | Fluctuating |
| S49 | L236P/N | EVA | 51 | 51 | 97.5 | 86.3 | Progressive |
| S54 | T416P/N | GJB2: 35delG/35delG | >90 | >90 | >100 | >100 | Stable |
| S55 | R79Q/N | ANSD | 81.3 | 83.8 | 71.3 | 70.0 | Stable |
| S104 | 1343-1344insAGTC/N | ANSD/EVA | 50 | 50 | 75.0 | >100 | Fluctuating/ Progressive |
| S110 | c.919_936delinsCCCCA/N | EVA | 55.0 | 53.8 | 83.8 | 120.0 | Fluctuating |
| S112 | IVS9+3A>G/N | EVA | 16.3 | 82.5 | 32.5 | 73.8 | Fluctuating |
| S150 | F335L/N | EVA | 45.0 | 47.5 | 15.0 | 16.3 | Fluctuating/Improved |
| S201 | c. 1001+1G>A/N |  | 90.0 | 90.0 | >100 | 101.3 | Stable |
| S258 | c.−103T>C |  | 40.0 | 30.0 | 42.5 | 41.3 | Fluctuating |
| S261 | D661E/N | GJB2:  V37I/V37I | 45 | 40 | 41.25 | 32.5 | Stable |
| S266 | S780F/N |  | 40.0 | 40.0 | 42.5 | 43.8 | Stable |
| S276 | c.−103T>C |  | 45.0 | 40.0 | 41.3 | 33.8 | Stable |
| S284 | Q514R/N |  | 60.0 | 60.0 | 63.8 | 57.5 | Fluctuating |
| S342 | L597S/N |  | 41.3 | 40.0 | 67.5 | 66.3 | Fluctuating |
| S348 | R776S/N | GJB2:  V271+E114G/N | 15 | 16.3 | 17.5 | 17.5 | Stable |
| S391 | IVS9+3A>G/N | Conductive | 46.3 | 46.3 | 72.5 | 70.0 | Progressive |
| S431 | R776C/N |  | 43.8 | 43.8 | 51.3 | 51.3 | Progressive/ Fluctuating |
| S508 | IVS6 + 2T>C/N | GJB2:  M34T/N | 72.5 | 72.5 | 87.5 | 81.3 | Progressive |
| S534 | L597S/N |  | 53.3 | 10.0 | 48.8 | 8.8 | Stable |
| S625 | L597S/N | GJB2:  T8M/V1531  EVA | 43.8 | 78.8 | 51.25 | 80 | Fluctuating |
| S708 | A429del/N |  | 47.5 | 47.5 | 48.8 | 48.8 | Fluctuating |
| S717 | R409H/N |  | 78.8 | 95.0 | 72.5 | >100 | Progressive |
| S809 | V358M/N |  | 56.3 | 56.3 | 38.75 | 38.75 | Stable |
| S814 | N324Y/N |  | 30 | 30 | 31.25 | 31.25 | Fluctuating |
|  | **Two mutations** |  |  |  |  |  |  |
| S249 | T416P/IVS11+1G>C |  | 71.3 | 61.3 | >100 | 81.3 | Progressive |
| S272 | c.1001+1G>A/G672E | EVA | 75.0 | 65.0 | 90.0 | 83.8 | Progressive/Fluctuating |
| S328 | L117F/L117F |  | 22.5 | 22.5 | 46.3 | 46.3 | Fluctuating |
| S389 | L236P/A429del |  | 50.0 | 30.0 | 83.8 | 65.0 | Progressive |
| S396 | Y375C /R470H |  | 109 | 109 | 93.75 | 93.75 | Stable |
| S483 | L236P/L597S |  | 63.8 | 63.8 | 70.0 | 70.0 | Fluctuating |
| S485 | S90L/S90L | EVA/CMV | 35.0 | 35.0 | 61.3 | 43.8 | Fluctuating |
| S495 | p.Leu450GlyX19/IVS1-2A>G |  | 50 | 60 | 60.0 | 91.3 | Progressive |
| S816 | Y375C/R470H |  | 100 | 107.5 | >100 | 112.5 | Stable |
| S485 | S90L/S90L | EVA/CMV | 35.0 | 35.0 | 61.3 | 43.8 | Fluctuating |
| S816 | Y375C/R470H |  | 100 | 107.5 | 100.0 | 112.5 | Stable |
