# Supplementary material for: Etiology and Audiological Outcomes at 3 Years for 364 Children in Australia
Source: PLoS One. 2013 Mar 28;8(3):e59624. doi: 10.1371/journal.pone.0059624 (PMC3610796; doi:10.1371/journal.pone.0059624)
Supplement: Table S3 — Hearing thresholds at time of diagnosis and evolution in children with hearing loss attributable to congenital CMV infection. (DOC) [file pone.0059624.s003.doc]

**Supplementary Table S3.** Hearing thresholds at time of diagnosis and evolution in children with hearing loss attributable to congenital CMV infection.

|  |  |  | Diagnosis | | 3 years of age | | Evolution |
| --- | --- | --- | --- | --- | --- | --- | --- |
| Participant | CMV | Other | HTL -left | HTL - right | HTL -left | HTL - right |  |
| S79 | Positive |  | 33.75 | 33.75 | 33.75 | 33.75 | Stable |
| S117 | Positive |  | 36.75 | 36.75 | 43.75 | 42.5 | Stable |
| S142 | Positive | *GJB2*: V371/N | 71.25 | 30 | 91.25 | 38.75 | Progressive |
| S212 | Positive |  | 63.75 | 90 | 68.75 | 83.75 | Stable |
| S239 | Positive |  | 116.25 | 116.25 | >100 | >100 | Stable |
| S252 | Positive | Perinatal trauma | 40 | 71.25 | 56.25 | 42.5 | Fluctuating/ Progressive |
| S308 | Positive |  | 51.25 | 93.75 | 48.75 | >110 | Progressive |
| S327 | Positive |  | 107.5 | 116.25 | >100 | >100 | Stable |
| S347 | Positive |  | 55 | 55 | 66.25 | 72.5 | Fluctuating |
| S372 | Positive |  | 117.5 | 111.25 | >100 | 111.25 | Stable |
| S374 | Positive | Perinatal trauma | 77.5 | 51.25 | >100 | >100 | Progressive |
| S425 | Positive |  | 88.75 | 90 | >100 | >100 | Stable |
| S426 | Positive | Meningitis | 102.5 | >100 | 102.5 | >100 | Stable |
| S428 | Positive |  | 56.25 | 56.25 | 36.25 | 33.75 | Stable |
| S434 | Positive | ANSD | >100 | 102 | >100 | 103.75 | Stable |
| S443 | Positive |  | 85 | 53.5 | 93.75 | 58.75 | Stable |
| S462 | Positive | *GJB2*: M34T/N | >90 | >90 | 120 | >100 | Stable |
| S463 | Positive |  | >100 | 30 | 115 | 33.75 | Stable |
| S471 | Positive |  | 52.5 | 52.5 | 71.25 | 72.5 | Stable |
| S485 | Positive | *SLC26A4*: S90L/S90L;  EVA | 35 | 35 | 61.25 | 43.75 | Fluctuating |
| S487 | Positive | CHARGE syndrome | 47.5 | 47.5 | 66.25 | 41.25 | Fluctuating |
| S521 | Positive |  | 48.75 | 12.5 | 48.75 | 12.5 | Stable |
| S528 | Positive |  | 68.75 | >100 | 62.5 | >100 | Stable |
| S605 | Positive |  | 76.25 | 116.25 | 107.5 | 125 | Stable |
| S611 | Positive |  | 42.5 | 42.5 | 42.5 | 43.75 | Fluctuating |
| S619 | Positive |  | 65 | 85 | 65 | 88.75 | Progressive |
| S802 | Positive |  | 115 | 115 | >100 | >100 | Stable |
| S803 | Positive |  | 91.25 | 91.25 | >100 | >100 | Stable |
| S811 | Positive | EVA | 117.5 | 117.5 | >100 | >100 | Stable |
| S832 | Positive | EVA | 85 | 85 | >100 | >100 | Stable |
