# Supplementary material for: Etiology and Audiological Outcomes at 3 Years for 364 Children in Australia
Source: PLoS One. 2013 Mar 28;8(3):e59624. doi: 10.1371/journal.pone.0059624 (PMC3610796; doi:10.1371/journal.pone.0059624)
Supplement: Table S4 — PolyPhen-2, SIFT BLink and SIFT Sequence predictions of novel or controversial amino acid changes on GJB2 and SLC26A4 gene product functions. (DOCX) [file pone.0059624.s004.docx]

**Supplementary Table S4.** PolyPhen-2, SIFT BLink and SIFT Sequence predictions of novel or controversial amino acid changes on *GJB2* and *SLC26A4* gene product functions.

| **Gene** | **Protein change** | **PolyPhen-2 (score)** | **SIFT BLink** | **SIFT Sequence** |
| --- | --- | --- | --- | --- |
|  |  |  |  |  |
| *GJB2* | K108N | Benign (0.001) | Tolerated | Affects protein function |
| *GJB2* | T186A | Probably damaging (0.999) | Not tolerated | Affects protein function |
|  |  |  |  |  |
| *SLC26A4* | R43H | Benign (0.001) | Tolerated | Tolerated |
| *SLC26A4* | R79Q | Probably damaging (0.977) | Tolerated | Tolerated |
| *SLC26A4* | F335L | Probably damaging (0.997) | Tolerated | Affects protein function |
| *SLC26A4* | L597S | Probably damaging (0.999) | Tolerated | Affects protein function |
| *SLC26A4* | S780F | Benign (0.005) | Not tolerated | Affects protein function |
